# Heart Rate Variability in Acute Myocardial Infarction: Results of the HeaRt-V-AMI Single-Center Cohort Study

**DOI:** 10.3390/jcdd11080254

**Published:** 2024-08-22

**Authors:** Crischentian Brinza, Mariana Floria, Dragos-Viorel Scripcariu, Alexandra Maria Covic, Adrian Covic, Iolanda Valentina Popa, Cristian Statescu, Alexandru Burlacu

**Affiliations:** 1 Institute of Cardiovascular Diseases “Prof. Dr. George I.M. Georgescu”, 700503 Iasi, Romania; crischentian.brinza@d.umfiasi.ro (C.B.); covic_maria-alexandra@d.umfiasi.ro (A.M.C.); cristian.statescu@umfiasi.ro (C.S.); 2 Faculty of Medicine, University of Medicine and Pharmacy “Grigore T Popa”, 700115 Iasi, Romania; floria.mariana@umfiasi.ro (M.F.); dragos-viorel.scripcariu@umfiasi.ro (D.-V.S.); adrian.covic@umfiasi.ro (A.C.); iolanda-valentina.g.popa@umfiasi.ro (I.V.P.); 3 Internal Medicine Clinic, “St. Spiridon” County Clinical Emergency Hospital Iasi, 700111 Iasi, Romania; 4 Nephrology Clinic, Dialysis, and Renal Transplant Center, “C.I. Parhon” University Hospital, 700503 Iasi, Romania

**Keywords:** heart rate variability, myocardial infarction, percutaneous coronary intervention, wearable technology, risk stratification

## Abstract

(1) Background: Heart rate variability (HRV) has been investigated in the context of ST-segment elevation myocardial infarction (STEMI). This study contributes to the field by assessing short-term HRV during primary percutaneous coronary intervention (PCI) using wearable technology, providing real-time insights into autonomic function. (2) Methods: This single-center, observational cohort study included 104 STEMI patients undergoing primary percutaneous coronary intervention (PCI). HRV parameters (including SDNN, RMSSD, pNN50, HF, SD1, and SD2/SD1 ratio) were measured using a wearable device (Empatica E4 wristband, CE certified). Measurements were taken throughout the entire duration of the primary PCI, as well as specifically during the initial 5 min and the final 5 min of the procedure. The association between HRV parameters and adverse outcomes, including in-hospital mortality and in-hospital major adverse cardiovascular events (MACE), were assessed. (3) Results: HRV parameters significantly decreased after myocardial revascularization, particularly SDNN, RMSSD, pNN50, HF, SD1, and SD2/SD1 ratio. Significant associations were found between reduced SD2/SD1 ratio, approximate entropy, and adverse outcomes, including increased in-hospital mortality and in-hospital MACE (respectively, *p* = 0.007, *p* = 0.017 and *p* = 0.006 and *p* = 0.005). The SD2/SD1 ratio was significantly lower in patients who died during the hospital stay (*p* = 0.008) compared to survivors. Approximate entropy was also significantly lower in deceased patients (*p* = 0.019). (4) Conclusions: Real-time HRV monitoring using wearable technology offers valuable data regarding dynamic physiological changes during primary PCI. Further studies are required to validate these preliminary results and to explore their potential implications for clinical practice.

## 1. Introduction

Ischemic heart disease, particularly ST-segment elevation myocardial infarction (STEMI), remains a leading cause of global morbidity and mortality [1]. Despite advancements in acute management of STEMI patients, there is a persistent need for accurate and individualized prognostic markers to better predict outcomes and tailor therapeutic strategy [2,3]. Traditional risk stratification methods might not capture the dynamic nature of patient’s risk, requiring an individualized approach to enhance the prognostic accuracy.

Heart rate variability (HRV), a measure of autonomic nervous system function, has been extensively studied and recognized as a valuable prognostic tool in cardiovascular disease, particularly in the context of STEMI. Numerous studies have demonstrated that alterations in HRV are associated with adverse outcomes, including increased mortality and major adverse cardiovascular events (MACE) [4,5,6]. Also, by capturing real-time physiological responses, HRV measurements offer individualized insights into the dynamic autonomic nervous system activity [7]. Nevertheless, the relationship between short-term and instantaneous HRV parameters and clinical and paraclinical variables in STEMI patients is not yet fully understood.

The clinical application of HRV has been limited by the use of 24 h electrocardiographic recordings, which could be impractical for real-time monitoring in acute settings. To address this limitation, wearable devices have been validated for their accuracy in measuring HRV, offering a practical alternative to traditional electrocardiographic recordings [8]. These devices facilitate the collection of HRV data in diverse settings, ranging from everyday activities to acute myocardial infarction (AMI) scenarios, thereby broadening the scope of HRV research and its applications. Additionally, wearables enable continuous monitoring, which is particularly beneficial for observing transient physiological changes in HRV parameters [8].

In a comprehensive overview, Shaffer and Ginsberg, identified and established norms for various HRV metrics, including short and ultra-short parameters [9]. These norms provide valuable benchmarks for interpreting HRV in a range of clinical settings. However, despite the broad applicability of these metrics, there remains a notable gap in the literature concerning the HRV measurements in STEMI patients during the acute phase [9]. A recent study examined HRV and heart rhythm complexity in 33 patients with inferior STEMI within one year following myocardial infarction [10]. The authors reported significant changes in this metrics over time, underscoring the potential of HRV in monitoring cardiovascular health [10].

The alterations in HRV during the initial hours following STEMI onset are still not well described, suggesting a need for further research to elucidate early responses in these patients. Capturing real-time physiological data facilitates a more personalized approach to patient care. Our study represents a key step in integrating wearable technology with clinical practice, aiming to improve prognostic accuracy and ultimately enhance patient outcomes in STEMI management.

The primary objective of this study was to assess the dynamic changes in various HRV parameters (time-domain, frequency-domain and non-linear measurements) during the acute phase of STEMI, and to establish baseline metrics and normative ranges for these parameters. Additionally, we sought to explore the association between real-time HRV measurements and adverse outcomes during hospitalization for STEMI.

## 2. Materials and Methods

This study was a single-center, observational cohort study conducted at the Institute of Cardiovascular Diseases “Prof. Dr. George I.M. Georgescu” in Iasi, Romania. Ethical approval was obtained from both the Institute’s Ethics Committee (approval date: 14 January 2022) and the University of Medicine and Pharmacy “Grigore T. Popa” Ethics Committee (approval number: 164/21.03.2022). Also, the protocol of this study was registered in the ClinicalTrials.gov database (NCT05098977). The results will be updated in the ClinicalTrials.gov database following the publication of this article, in accordance with the appropriate regulatory requirements. All participants were informed about the objectives of this study, potential risks and benefits, and written informed consent was obtained from each participant. Confidentiality and data protection were ensured, as all collected data were anonymized and stored in a secure database.

### 2.1. Study Population

Consecutive patients presenting with STEMI in sinus rhythm, referred for primary percutaneous coronary intervention (PCI) within 12 h from symptoms onset were enrolled. Inclusion criteria included (1) age ≥ 18 years, (2) STEMI diagnosis in the first 12 h from symptoms onset, and (3) the ability to provide informed consent.

Exclusion criteria encompassed conditions interfering with RR intervals and HRV parameters: (1) atrioventricular block of any degree or known sinus node dysfunction, (2) atrial fibrillation, (3) paced ventricular rhythm, (4) patients treated with positive inotropic or chronotropic drugs, (5) history of myocardial infarction or revascularization, and (6) inability to sign the informed consent.

### 2.2. HRV Measurement

HRV parameters were measured during primary PCI using a wearable device approved for medical use (Empatica E4 wristband, CE certified). Measurements were taken throughout the entire duration of the primary PCI, as well as specifically during the initial 5 min and the final 5 min of the procedure, to capture dynamic short-term physiological responses. The following time-domain HRV parameters were calculated: the standard deviation of all NN intervals (SDNN), the standard deviation of the average NN interval over short time divisions (SDANN), the HRV triangular index, the square root of the mean squared differences of consecutive NN intervals (RMSSD), the number of pairs of successive NN (R-R) intervals that differ by more than 50 ms (NN50), and the proportion of NN50 divided by the total number of NN (R-R) intervals (pNN50). Frequency-domain measurements included low-frequency power (LF), very-low-frequency power (VLF), high-frequency power (HF), as well as LF/HF ratio. Additionally, the following non-linear parameters were evaluated: SD1, SD2, SD2/SD1, and approximate entropy.

### 2.3. Study Outcomes

The primary outcome of this study was to assess the dynamic changes in various short-term HRV parameters (time-domain, frequency-domain, and non-linear measurements) during the acute phase of STEMI, and to establish baseline metrics and normative ranges for these parameters. Additionally, we explored the relationship between real-time HRV measurements and other clinical and paraclinical variables in the first hours from symptoms onset.

Furthermore, we analyzed the association between all HRV parameters and in-hospital adverse outcomes, including all-cause and cardiovascular mortality, as well as MACE. MACE was defined as all-cause and cardiovascular mortality, fatal and non-fatal myocardial infarction, unplanned target vessel revascularization, and ischemic or hemorrhagic stroke.

### 2.4. Statistical Analysis

Statistical analysis was conducted using SPSS software version 26.0 (IBM SPSS Statistics, Armonk, NY, USA), and R software version 4.2.1 (R Foundation for Statistical Computing, Vienna, Austria; with the following packages: ‘dplyr’ for data manipulation, ‘ggplot2’ for visualizations, and ‘stats’ for statistical tests). Also, Kubios HRV Standard software (Kubios Oy, Kuopio, Finland) was used to analyze extracted interbeat interval data from the wristband and to calculate all HRV parameters. To ensure the accuracy of HRV parameters, any artifacts in the raw data were corrected using a very low threshold algorithm (0.45 s) in Kubios HRV software, minimizing the potential for significant distortions in HRV measurement.

Missing data were handled using the standard multiple imputation procedure in SPSS, generating five imputed datasets. The results reported in the manuscript are based on the pooled estimates across all imputed datasets, following SPSS’s automatic pooling of results from multiple imputations. Notably, key variables such as all HRV parameters and outcomes had no missing data. The distribution of the data was assessed using the Shapiro–Wilk test. Non-parametric tests, such as the Wilcoxon signed-rank test and the Mann–Whitney U test, were used due to the non-normal distribution of HRV parameters, ensuring robustness and reliability in the statistical analysis. The Wilcoxon signed-rank test was employed to analyze dynamic changes in HRV parameters over different time points. Additionally, we performed ROC analysis to predict adverse events during hospitalization. ROC analysis was conducted using logistic regression as the classification model to assess the predictive power of HRV parameters.

All statistical tests were two-tailed, and a *p*-value of <0.05 was considered statistically significant. The analyses were conducted in accordance with relevant guidelines and best practices for statistical analysis in medical research.

## 3. Results

Between April 2022 and December 2023, a total of 104 patients with STEMI who underwent primary PCI were included in this study. Among them, 70.2% were male and 29.8% were female, with a mean age of 60.64 ± 13.36 years. Baseline demographics and characteristics of patients were displayed in Table 1. The median time to PCI was 8 h with an interquartile range (IQR) of 6.0 to 10.25 h. Regarding cardiovascular risk factors, 58.65% of the patients were smokers, 55.8% had arterial hypertension, and 21.15% had diabetes mellitus. Additionally, 15.4% had a history of ischemic heart disease, 5.8% had experienced a previous stroke, 2.9% had peripheral artery disease, and 2.9% had chronic obstructive pulmonary disease. Also, 23.1% patients were treated with beta-blockers prior to the index hospitalization.

Most patients presented with inferior STEMI (50%), followed by extensive anterior STEMI (24%), with a median admission left ventricular ejection fraction (LVEF) of 40%. Additionally, the majority of patients were in Killip class I (66.3%) and class II (27.9%). Regarding coronary artery disease severity, 46.2% of patients had single-vessel disease, 31.7% had two-vessel disease, and 22.1% had three-vessel disease. The most frequent culprit artery was the left anterior descending (LAD) artery (48.1%), followed by the right coronary artery (RCA, 35.6%). Final TIMI flow grade 3 was achieved in 88.5% of patients (Table 2).

HRV parameters were measured throughout the entire PCI procedure. These measurements were also divided into two 5 min segments: one at the beginning and one at the end of the procedure, to capture the potential effects of percutaneous myocardial revascularization. All measurements are presented in Table 3. Notably, all HRV parameters (time-domain, frequency-domain, and non-linear measurements) tended to decrease in the last 5 min segment compared to the first 5 min segment, although not all changes achieved statistical significance. Mortality occurred in five patients (4.8%), and MACE occurred in six cases (5.8%). Cardiac arrest during PCI was observed in two patients (1.9%), and ventricular arrhythmias were documented in 12 patients (11.5%). The median ICU stay was 3.0 days (Supplementary Table S1).

Among time-domain measurements, a significant decrease following PCI was noted for SDNN, RMSSD, and pNN50 (all changes were displayed in Table 4; the values in the second column represent the Z-scores obtained from the Wilcoxon signed-rank test). Similarly, in the case of frequency-domain parameters, the decrease in HF reached statistical significance, while the other parameters remained relatively unchanged when comparing the first and last 5 min of PCI. Additionally, non-linear parameters also decreased shortly after PCI, with SD1 and the SD2/SD1 ratio showing statistically significant changes (Supplementary Figures S1–S3).

Compared to patients with RCA as culprit artery, those with LAD as culprit artery tended to have lower HRV parameter values (Supplementary Table S2). In time-domain measurements, SDNN, RMSSD, pNN50, and the RR triangular index were significantly lower in patients with LAD as the culprit artery. In case of frequency-domain parameters, VLF, LF (ms^2^, log), and HF (ms^2^, log) were significantly higher in RCA-culprit patients, while the LF/HF ratio was similar in both groups. Additionally, SD1, SD2, and the SD2/SD1 ratio were lower in LAD-culprit as compared to RCA-culprit patients.

Concerning in-hospital mortality, the SD2/SD1 ratio was significantly lower in deceased patients compared to survivors throughout the entire PCI procedure, including measurements taken in 5 min segments (*p* = 0.008). Additionally, the approximate entropy measure for the entire PCI duration was significantly lower in deceased patients (*p* = 0.019), although the 5 min segments did not reach statistical significance (Table 5). In the correlation analysis using the Spearman test, LF (log), SD2/SD1, and approximate entropy were significantly correlated with in-hospital mortality (*p* = 0.048, *p* = 0.007, and *p* = 0.017, respectively) (Supplementary Table S3).

In order to develop a predictive model for the in-hospital mortality, we selected the features that were significantly associated with the outcome, as resulted from Table 5. As such, only two variables were found to significantly differ between mortality groups (deceased versus survivors): SD2/SD1 ratio and approximate entropy.

**Figure 1:**
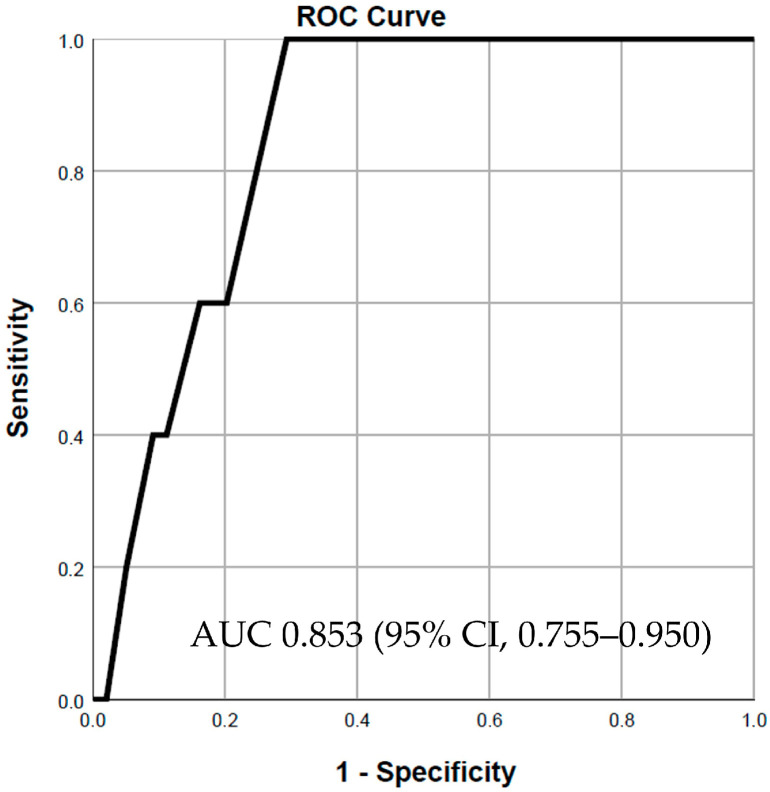
SD2/SD1 ratio for in-hospital mortality prediction.

**Figure 2:**
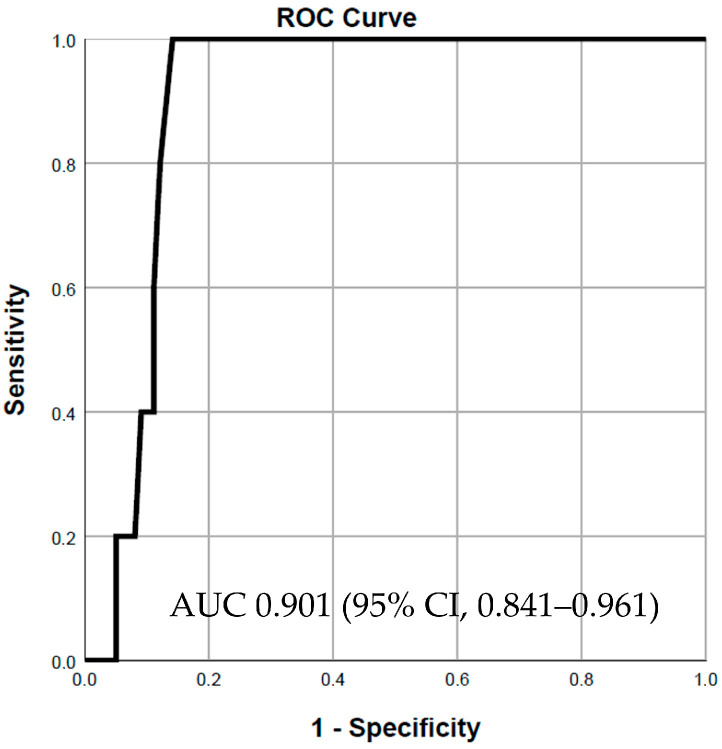
SD2/SD1 ratio and approximate entropy for in-hospital mortality prediction.

Both selected variables were successively incorporated into a logistic regression model to predict in-hospital mortality. Firstly, the SD2/SD1 ratio demonstrated good predictive power, with an area under the curve (AUC) of 0.853 (95% CI, 0.755–0.950, *p* = 0.008) (Figure 1). Adding approximate entropy to the model increased the predictive power, resulting in an AUC of 0.901 (95% CI, 0.841–0.961, *p* = 0.003) (Figure 2). However, including LVEF in the final model to assess its impact on predictive power did not lead to a statistically significant change (chi-square test, *p* = 0.234). Notably, for patients with severe systolic dysfunction (LVEF ≤ 30%), the predictive power of the combined model using the SD2/SD1 ratio and approximate entropy remained strong, with an AUC of 0.816 (95% CI, 0.641–0.990) (Supplementary Figure S4).

Patients who experienced MACE and ventricular arrhythmias exhibited altered non-linear HRV parameters compared to those without adverse events. Specifically, patients with MACE during hospitalization had significantly lower SD2/SD1 ratio and approximate entropy values (*p* = 0.006 and *p* = 0.005, respectively). Similarly, ventricular arrhythmias were associated with a significantly reduced SD2/SD1 ratio (*p* = 0.002) (Supplementary Table S4).

## 4. Discussion

Our study is the first prospective investigation to evaluate dynamic changes in HRV parameters during the acute phase of STEMI using real-time measurements from a CE-marked wearable device (Empatica E4 wristband). This innovative approach enabled continuous monitoring of patients during the primary PCI procedure, offering valuable insights into autonomic nervous system activity during this critical period.

The primary novelty of our study lies in the use of real-time HRV monitoring with a wearable device, which allowed continuous data collection throughout the entire PCI procedure. Previous studies have typically relied on 24 h electrocardiographic recordings, which are impractical for real-time monitoring in acute settings [11,12]. The wearable device used in our study is validated for medical use, ensuring the accuracy and reliability of the HRV measurements obtained [8]. By capturing short-term physiological responses, one could assess transient physiological changes, providing data regarding autonomic responses to acute myocardial infarction and subsequent revascularization.

The present study has several key strengths.

Our findings revealed a significant decrease in the majority of HRV parameters shortly after myocardial revascularization, including SDNN, RMSSD, pNN50, HF, SD1, and the SD2/SD1 ratio, underscoring the impact of PCI on autonomic function. Moreover, our analysis highlighted different HRV values in patients with LAD as the culprit artery compared to those with RCA as the culprit artery. LAD-culprit patients showed significantly lower values in parameters such as SDNN, RMSSD, and pNN50.

Furthermore, we evaluated adverse in-hospital outcomes such as cardiovascular mortality, MACE, and ventricular arrhythmias, establishing correlations with HRV metrics. Notably, reduced values of SD2/SD1 and approximate entropy were significantly associated with increased in-hospital mortality. Similarly, a lower SD2/SD1 ratio was linked to a higher incidence of ventricular arrhythmias. In case of patients who experienced MACE, both SD2/SD1 and approximate entropy were significantly decreased.

Chakko et al. [12], reported similar findings regarding HRV parameter alterations following myocardial reperfusion. They observed a significant decrease in HRV parameters, measured over 24 h, following myocardial reperfusion primarily achieved through thrombolysis. This decrease was attributed to acute autonomic imbalance induced by myocardial reperfusion, underscoring the immediate impact of revascularization on autonomic function [12].

In an earlier study, Huikuri et al. [13], found that HRV metrics were linked to an increased risk of mortality and arrhythmic events. However, PCI was performed in only 25% of patients presenting with AMI, contrasting with contemporary practice where PCI is the standard of care [13]. Therefore, the data on HRV monitoring in these patients needed updating, particularly with real-time derived parameters, to better stratify the risk of adverse events.

A recent study examined the impact of immediate versus delayed stenting strategies (high thrombus burden) on short-term HRV in STEMI patients undergoing primary PCI [14]. The delayed stenting approach was linked to improved postoperative cardiac electrical stability, indicated by fewer premature ventricular contractions and higher HRV parameters such as SDNN and HF. This study reinforces the utility of HRV assessment in STEMI patients, aiding in the identification of those at higher risk for adverse events [14].

Prognostic stratification in STEMI patients is a complex process that involves multiple parameters, including advanced imaging techniques such as early cardiac magnetic resonance (CMR). Recent studies highlighted the role of CMR in assessing myocardial inflammation, which has been linked to patient prognosis [15]. HRV could serve as a valuable additional parameter, particularly in evaluating the inflammatory and sympathetic responses in STEMI patients. The integration of HRV with imaging parameters might enhance the prediction of outcomes and tailor therapeutic strategies. Further studies are needed to explore the potential of combining HRV with advanced imaging modalities in this clinical context.

While our study provides novel insights into the real-time measurement of HRV parameters during PCI, it also has several limitations.

Firstly, the single-center design may limit the generalizability of our findings to other settings or populations. Additionally, the relatively small sample size may reduce the statistical power to detect more subtle differences or associations. Furthermore, the observational nature of this study precludes any conclusions about causality between HRV changes and clinical outcomes. Also, we explored the associations between HRV parameters and short-term adverse outcomes without applying a formal correction for multiple comparisons, given the exploratory nature of this study. Future research will include more stringent statistical controls to validate the findings. Due to the small number of adverse events, we analyzed the entire dataset to maximize statistical power and did not apply validation techniques, as splitting the data could have compromised the reliability of our findings. Given the limited sample size and the small number of events, performing a multivariate analysis to adjust for potential confounding factors was not feasible. We consider incorporating adjustment for confounding factors in subsequent studies, particularly when larger datasets will be available and when the focus will move from exploratory to confirmatory analyses. Future multicenter studies with larger sample sizes are required to validate our findings and to incorporate HRV metrics into future risk scores for STEMI patients.

Our study highlights the significant advantages of real-time HRV monitoring during PCI in STEMI patients, demonstrating its utility in improving risk stratification and patient outcomes. These promising results support the integration of HRV metrics into routine clinical practice, fostering better management and prognosis for STEMI patients. While our study constitutes a foundational background for the utility of HRV analysis in STEMI patients, future multicenter studies with larger sample sizes are required to validate our findings.

## 5. Conclusions

The present study highlights the significant potential of real-time HRV monitoring using wearable technology during the acute phase of STEMI. This approach could offer a new perspective for enhancing patient care through individualized and dynamic risk stratification. The primary findings illustrate that PCI significantly impacts HRV parameters, with a significant decrease in key metrics such as SDNN, RMSSD, and pNN50, highlighting the acute autonomic responses to myocardial revascularization. The association between reduced HRV parameters, particularly the SD2/SD1 ratio and approximate entropy, and adverse in-hospital outcomes, underscores the prognostic utility of HRV monitoring. The integration of HRV metrics into routine clinical practice, facilitated by wearable devices, could improve the risk stratification in STEMI patients.

## Figures and Tables

**Table 1:** Baseline demographics and characteristics of included patients.

Characteristics	Overall (*n* = 104)
Age, mean ± SD, years	60.64 ± 13.36
Women, n (%)	31 (29.8)
BMI, mean ± SD, kg/m^2^	28.42 ± 5.69
BSA, mean ± SD, m^2^	1.91 ± 0.25
Smoking, n (%)	61 (58.65)
Alcohol consumption, n (%)	17 (16.35)
Sedentarism, n (%)	96 (92.31)
Time to PCI, median (IQR), hours	8.0 (6.0–10.25)
History of IHD, n (%)	16 (15.4)
Arterial hypertension, n (%)	58 (55.8)
Previous stroke, n (%)	6 (5.8)
Diabetes mellitus	22 (21.15)
PAD, n (%)	3 (2.9)
COPD, n (%)	3 (2.9)
Chronic hepatitis, n (%)	1 (1.0)
Prior beta-blocker, n (%)	24 (23.1)
Killip class	
Class I, n (%)	69 (66.3)
Class II, n (%)	29 (27.9)
Class III, n (%)	6 (5.8)
STEMI territory	
Anterior (V3, V4), n (%)	3 (2.9)
Anteroseptal (V1–V4), n (%)	23 (22.1)
Anterolateral (V3–V6), n (%)	1 (1.0)
Extensive anterior (V1–V6), n (%)	25 (24.0)
Inferior (II, III, aVF)	52 (50.0)
SBP, mean ± SD, mmHg	145.85 ± 21.85
eGFR, mean ± SD, mL/min/1.73 m^2^	82.96 ± 23.56
CK-MB, admission, median (IQR), U/L	48.0 (23.0–89.2)
CK-MB, peak, median (IQR), U/L	188 (87.0–288.5)
AST, median (IQR), U/L	56 (36.0–102.5)
ALT, median (IQR), U/L	34.0 (25.0–51.7)
CRP, median (IQR), mg/dL	29.9 (12.5–65.7)
Fibrinogen, median (IQR), mg/dL	431.0 (371.7–517.0)
LDL, mean ± SD, mg/dL	136.1 ± 53.5
Triglycerides, mean ± SD, mg/dL	165.8 ± 108.9
Blood glucose, mean ± SD, mg/dL	162.8 ± 60.8
LVEF, admission, median (IQR), %	40.0 (34.7–45.0)
LVEF, discharge, median (IQR), %	40.0 (35.0–47.0)
E/A	
<1, n (%)	63 (60.6)
>2, n (%)	5 (4.8)

ALT = alanine aminotransferase; AST = aspartate aminotransferase; BMI = body mass index; BSA = body surface area; COPD = chronic obstructive pulmonary disease; CRP = C reactive protein; eGFR = estimated glomerular filtration rate; IHD = ischemic heart disease; LVEF = left ventricular ejection fraction; PAD = peripheral artery disease; PCI = percutaneous coronary intervention; SBP = systolic blood pressure STEMI = ST-segment elevation myocardial infarction.

**Table 2:** Angiographic findings and PCI characteristics.

PCI Characteristics	Overall (n = 104)
Coronary lesions	
Single artery, n (%)	48 (46.2)
Two arteries, n (%)	33 (31.7)
Three arteries, n (%)	23 (22.1)
Culprit artery	
LAD, n (%)	50 (48.1)
LCX, n (%)	14 (13.5)
RCA, n (%)	37 (35.6)
LM, n (%)	2 (1.9)
DG, n (%)	1 (1.0)
LM disease, n (%)	4 (3.8)
Initial TIMI flow	
TIMI 0, n (%)	57 (54.8)
TIMI 1, n (%)	10 (9.6)
TIMI 2, n (%)	18 (17.3)
TIMI 3, n (%)	19 (18.3)
Final TIMI flow	
TIMI 0, n (%)	0 (0)
TIMI 1, n (%)	1 (1.0)
TIMI 2, n (%)	10 (9.6)
TIMI 3, n (%)	92 (88.5)
Contrast media, mean ± SD, mL	204.8 ± 51.4

DG = diagonal artery; LAD = left anterior descending artery; LCX = left circumflex artery; LM = left main; PCI = percutaneous coronary intervention; RCA = right coronary artery; TIMI = thrombolysis in myocardial infarction.

**Table 3:** HRV parameters (entire PCI duration, first and last 5 min).

HRV Parameters	Overall	First 5 min	Last 5 min
SDNN, median (IQR), ms	29.2 (20.87–40.85)	29.5 (20.7–43.7)	28.15 (19.1–39.0)
RMSSD, median (IQR), ms	32.5 (25.7–46.4)	36.7 (25.2–52.0)	29.8 (22.3–45.5)
NN50, median (IQR), beats	89.0 (36.0–159.5)	28.5 (11.0–51.7)	18.5 (7.7–45.7)
pNN50, median (IQR), %	7.5 (3.1–17.7)	10.3 (3.0–20.7)	4.5 (2.0–17.9)
RR triangular index, median (IQR), ms	6.3 (5.3–8.8)	6.2 (4.8–8.7)	6.1 (4.5–8.0)
VLF, median (IQR), ms^2^	75.8 (35.7–142.4)	66.0 (19.1–130.5)	40.2 (22.5–154.6)
VLF, median (IQR), log	4.3 (3.5–4.9)	4.1 (2.9–4.8)	3.6 (3.0–5.0)
LF, median (IQR), ms^2^	372.9 (175.3–654.5)	365.2 (108.2–712.5)	359.2 (101.0–751.5)
LF, median (IQR), log	5.9 (5.1–6.4)	5.8 (4.6–6.5)	5.8 (4.5–6.6)
LF, median (IQR), n.u.	66.9 (59.8–74.8)	63.9 (54.9–73.5)	65.3 (56.0–75.4)
HF, median (IQR), ms^2^	168.5 (96.1–310.0)	188.0 (71.4–332.5)	155.5 (59.4–290.3)
HF, median (IQR), log	5.1 (4.5–5.7)	5.2 (4.2–5.8)	5.0 (4.0–5.6)
HF, median (IQR), n.u.	32.7 (25.0–39.8)	35.1 (25.8–44.5)	34.3 (24.4–43.2)
LF/HF median (IQR)	2.0 (1.4–2.9)	1.7 (1.2–2.8)	1.9 (1.2–3.0)
SD1, median (IQR), ms	23.0 (18.2–32.9)	26.0 (17.8–37.0)	21.1 (15.8–32.2)
SD2, median (IQR), ms	33.3 (22.2–44.5)	32.7 (22.2–47.8)	32.2 (21.4–46.3)
SD2/SD1, median (IQR)	1.3 (1.0–1.5)	1.2 (0.9–1.5)	1.3 (1.0–1.6)
ApEn, median (IQR)	1.3 (1.2–1.4)	1.0 (0.9–1.1)	1.0 (0.9–1.1)

ApEn = approximate entropy; HF = power in high-frequency range; LF = power in low-frequency range; NN50 = the number of pairs of successive NN (R-R) intervals that differ by more than 50 ms; pNN50 = the proportion of NN50 divided by the total number of NN (R-R) intervals; RMSSD = the square root of the mean squared differences of consecutive NN intervals; SDANN = the standard deviation of the average NN interval over short time divisions; SDNN = the standard deviation of all NN intervals; VLF = power in very-low-frequency range.

**Table 4:** HRV parameters modification during the PCI procedure.

HRV Parameters	First vs. Last 5 min of PCI	*p*-Value
SDNN, ms	Z −2.155	*p* = 0.031
RMSSD, median (IQR), ms	Z −3.436	*p* < 0.001
NN50, median (IQR), beats	Z −1.807	*p* = 0.071
pNN50, median (IQR), %	Z −3.043	*p* = 0.002
RR triangular index, median (IQR), ms	Z −1.786	*p* = 0.075
VLF, median (IQR), ms^2^	Z −0.417	*p* = 0.677
VLF, median (IQR), log	Z −0.581	*p* = 0.580
LF, median (IQR), ms^2^	Z −0.987	*p* = 0.324
LF, median (IQR), log	Z −0.737	*p* = 0.447
LF, median (IQR), n.u.	Z −0.858	*p* = 0.395
HF, median (IQR), ms^2^	Z −2.289	*p* = 0.022
HF, median (IQR), log	Z −2.116	*p* = 0.033
HF, median (IQR), n.u.	Z −0.914	*p* = 0.358
LF/HF median (IQR)	Z −1.199	*p* = 0.235
SD1, median (IQR), ms	Z −3.446	*p* < 0.001
SD2, median (IQR), ms	Z −1.850	*p* = 0.064
SD2/SD1, median (IQR)	Z −3.748	*p* < 0.001
ApEn, median (IQR)	Z −1.931	*p* = 0.055

ApEn = approximate entropy; HF = power in high-frequency range; LF = power in low-frequency range; NN50 = the number of pairs of successive NN (R-R) intervals that differ by more than 50 ms; pNN50 = the proportion of NN50 divided by the total number of NN (R-R) intervals; RMSSD = the square root of the mean squared differences of consecutive NN intervals; SDANN = the standard deviation of the average NN interval over short time divisions; SDNN = the standard deviation of all NN intervals; VLF = power in very-low-frequency range.

**Table 5:** Comparative HRV parameters in survivors and deceased patients.

HRV Parameters	Deceased	Survivors	*p*-Value
HRV during entire PCI duration			
SDNN, median (IQR), ms	23.9 (20.2–35.6)	29.2 (21.2–41.1)	*p* = 0.806
RMSSD, median (IQR), ms	38.9 (29.8–49.5)	32.3 (25.1–46.1)	*p* = 0.361
NN50, median (IQR), beats	102.0 (51.0–106.0)	86.5 (32.2–167.7)	*p* = 0.981
pNN50, median (IQR), %	14.1 (7.3–22.5)	7.2 (2.9–17.4)	*p* = 0.205
RR triangular index, median (IQR), ms	5.7 (4.9–6.3)	6.3 (5.3–8.8)	*p* = 0.447
VLF, median (IQR), ms^2^	33.3 (29.2–76.2)	75.8 (37.7–142.6)	*p* = 0.155
VLF, median (IQR), log	3.5 (3.3–4.3)	4.3 (3.6–4.9)	*p* = 0.157
LF, median (IQR), ms^2^	178.5 (113.5–194.5)	391.0 (178.2–681.7)	*p* = 0.064
LF, median (IQR), log	5.1 (4.7–5.2)	5.9 (5.1–6.5)	*p* = 0.052
LF, median (IQR), n.u.	63.8 (58.0–70.4)	66.9 (59.9–74.8)	*p* = 0.369
HF, median (IQR), ms^2^	100.4 (81.6–106.4)	178.7 (96.5–323.5)	*p* = 0.108
HF, median (IQR), log	4.6 (4.4–4.6)	5.1 (4.5–5.7)	*p* = 0.106
HF, median (IQR), n.u.	35.9 (29.4–41.7)	32.7 (25.0–39.7)	*p* = 0.373
LF/HF median (IQR)	1.7 (1.3–2.3)	2.0 (1.5–2.9)	*p* = 0.352
SD1, median (IQR), ms	27.5 (21.1–35.1)	22.8 (17.7–32.6)	*p* = 0.349
SD2, median (IQR), ms	19.7 (19.2–36.3)	33.3 (22.7–45.2)	*p* = 0.240
SD2/SD1, median (IQR)	0.9 (0.8–1.0)	1.3 (1.0–1.6)	*p* = 0.008
ApEn, median (IQR)	1.2 (1.0–1.2)	1.3 (1.2–1.4)	*p* = 0.019
HRV in the first 5 min of PCI			
SDNN, median (IQR), ms	32.3 (20.5–44.8)	29.3 (21.0–43.5)	*p* = 0.841
RMSSD, median (IQR), ms	51.9 (32.0–69.0)	36.3 (24.3–51.5)	*p* = 0.240
NN50, median (IQR), beats	23.0 (17.0–33.0)	28.5 (11.0–50.7)	*p* = 0.847
pNN50, median (IQR), %	26.1 (7.4–26.6)	9.1 (2.8–20.5)	*p* = 0.174
RR triangular index, median (IQR), ms	5.1 (4.8–6.4)	6.2 (4.8–9.1)	*p* = 0.256
VLF, median (IQR), ms^2^	24.0 (20.5–88.7)	66.0 (18.9–136.7)	*p* = 0.357
VLF, median (IQR), log	3.1 (3.0–4.4)	4.1 (2.9–4.8)	*p* = 0.365
LF, median (IQR), ms^2^	139.7 (22.7–252.3)	387.5 (110.9–744.7)	*p* = 0.056
LF, median (IQR), log	4.9 (3.1–5.5)	5.8 (4.6–6.5)	*p* = 0.067
LF, median (IQR), n.u.	58.1 (53.1–59.8)	65.3 (55.0–73.8)	*p* = 0.252
HF, median (IQR), ms^2^	93.3 (37.4–125.8)	194.7 (73.3–341.1)	*p* = 0.099
HF, median (IQR), log	4.5 (3.6–4.8)	5.2 (4.2–5.8)	*p* = 0.106
HF, median (IQR), n.u.	41.7 (39.9–46.6)	34.0 (25.8–44.3)	*p* = 0.216
LF/HF median (IQR)	1.3 (1.1–1.4)	1.9 (1.2–2.8)	*p* = 0.199
SD1, median (IQR), ms	36.7 (22.7–49.1)	25.7 (17.2–36.6)	*p* = 0.234
SD2, median (IQR), ms	27.2 (18.2–39.5)	32.7 (22.4–48.0)	*p* = 0.499
SD2/SD1, median (IQR)	0.8 (0.7–0.8)	1.2 (1.0–1.5)	*p* = 0.006
ApEn, median (IQR)	1.0 (0.6–1.0)	1.0 (0.9–1.1)	*p* = 0.188
HRV in the last 5 min of PCI			
SDNN, median (IQR), ms	24.3 (19.3–44.7)	28.4 (19.1–38.7)	*p* = 0.944
RMSSD, median (IQR), ms	34.2 (28.2–45.2)	29.8 (21.9–45.7)	*p* = 0.429
NN50, median (IQR), beats	39.0 (18.0–40.0)	18.0 (7.0–47.2)	*p* = 0.333
pNN50, median (IQR), %	10.2 (4.0–21.9)	4.5 (1.8–17.4)	*p* = 0.246
RR triangular index, median (IQR), ms	4.7 (4.3–7.1)	6.1 (4.5–8.0)	*p* = 0.580
VLF, median (IQR), ms^2^	35.5 (18.7–77.9)	40.2 (23.2–155.0)	*p* = 0.489
VLF, median (IQR), log	3.5 (2.9–4.3)	3.69 (3.1–5.0)	*p* = 0.456
LF, median (IQR), ms^2^	400.4 (81.8–424.4)	337.1 (106.5–794.2)	*p* = 0.377
LF, median (IQR), log	5.9 (4.4–6.0)	5.7 (4.6–6.6)	*p* = 0.352
LF, median (IQR), n.u.	72.9 (51.0–74.6)	65.1 (56.2–75.0)	*p* = 0.932
HF, median (IQR), ms^2^	129.6 (78.3–143.6)	168.0 (59.3–328.7)	*p* = 0.279
HF, median (IQR), log	4.8 (4.3–4.9)	5.1 (4.0–5.6)	*p* = 0.262
HF, median (IQR), n.u.	26.8 (25.2–48.8)	34.4 (24.8–43.0)	*p* = 0.944
LF/HF median (IQR)	2.7 (1.0–2.9)	1.8 (1.3–2.9)	*p* = 0.938
SD1, median (IQR), ms	24.2 (19.9–32.0)	21.1 (15.5–32.4)	*p* = 0.429
SD2, median (IQR), ms	24.4 (18.8–33.6)	32.3 (21.4–46.4)	*p* = 0.329
SD2/SD1, median (IQR)	1.0 (0.9–1.0)	1.3 (1.0–1.6)	*p* = 0.018
ApEn, median (IQR)	1.0 (0.8–1.0)	1.0 (1.0–1.1)	*p* = 0.165

ApEn = approximate entropy; HF = power in high-frequency range; LF = power in low-frequency range; NN50 = the number of pairs of successive NN (R-R) intervals that differ by more than 50 ms; pNN50 = the proportion of NN50 divided by the total number of NN (R-R) intervals; RMSSD = the square root of the mean squared differences of consecutive NN intervals; SDANN = the standard deviation of the average NN interval over short time divisions; SDNN = the standard deviation of all NN intervals; VLF = power in very-low-frequency range.

## Data Availability

The raw data supporting the conclusions of this article will be made available by the authors on request.

## Supplementary Materials

The following supporting information can be downloaded at: https://www.mdpi.com/article/10.3390/jcdd11080254/s1, Figure S1: Time-domain parameters during PCI; Figure S2: Frequency-domain parameters during PCI; Figure S3: Non-linear parameters during PCI; Figure S4: SD2SD1 and ApEn mortality low FE; Table S1: In-hospital outcomes; Table S2: HRV in LAD vs. RCA; Table S3: Mortality correlation analysis; Table S4: HRV and MACE and VA.
