# Supplementary material for: Heart Rate Variability in Acute Myocardial Infarction: Results of the HeaRt-V-AMI Single-Center Cohort Study
Source: J Cardiovasc Dev Dis. 2024 Aug 22;11(8):254. doi: 10.3390/jcdd11080254 (PMC11355001; doi:10.3390/jcdd11080254)
Supplement: Supplementary file 1 [file jcdd-11-00254-s001.zip › Table S1. In-hospital outcomes.pdf]

Table S1. In-hospital outcomes.

In-hospital outcomes	Overall (n = 104)
MACE, n (%)	6 (5.8)
Mortality, n (%)	5 (4.8)
CA during PCI, n (%)	2 (1.9)
CIN, n (%)	13 (12.5)
Ventricular arrhythmias, n (%)	12 (11.5)
ICU stay, median (IQR), days	3.0 (2.0-3.0)
CA = cardiac arrest; CIN = contrast induced nephropathy; ICU = intensive care unit; MACE = major adverse cardiovascular events	
