# Supplementary material for: Heart Rate Variability in Acute Myocardial Infarction: Results of the HeaRt-V-AMI Single-Center Cohort Study
Source: J Cardiovasc Dev Dis. 2024 Aug 22;11(8):254. doi: 10.3390/jcdd11080254 (PMC11355001; doi:10.3390/jcdd11080254)
Supplement: Supplementary file 1 [file jcdd-11-00254-s001.zip › Table S2. HRV in LAD vs RCA.pdf]

Table S2. Comparative HRV parameters in LAD and RCA culprit arteries.

HRV parameters	LAD-culprit	RCA-culprit	P-value
SDNN, median (IQR), ms	25.6 (19.4-35.0)	35.4 (25.7-53.5)	$p < 0.001$
RMSSD, median (IQR), ms	30.0 (24.0-43.0)	38.9 (28.9-56.0)	$p = 0.010$
NN50, median (IQR), beats	65.5 (31.5-136.7)	112.0 (47.0-170.0)	$p = 0.136$
pNN50, median (IQR), %	5.5 (2.6-14.0)	10.7 (4.4-27.4)	$p = 0.017$
RR triangular index, median (IQR), ms	5.9 (4.9-8.1)	7.8 (6.0-10.4)	$p = 0.002$
VLF, median (IQR), ms^2	60.2 (26.1-96.6)	126.3 (63.1-257.1)	$p < 0.001$
VLF, median (IQR), log	4.0 (3.2-4.5)	4.8 (4.1-5.5)	$p < 0.001$
LF, median (IQR), ms^2	290.4 (139.4-526.4)	575.4 (289.8-1240.8)	$p < 0.001$
LF, median (IQR), log	5.6 (4.9-6.1)	6.3 (5.6-7.1)	$p < 0.001$
LF, median (IQR), n.u.	66.6 (61.7-73.7)	73.8 (59.7-77.6)	$p = 0.196$
HF, median (IQR), ms^2	137.4 (72.1-243.5)	244.8 (110.9-606.6)	$p = 0.002$
HF, median (IQR), log	4.8 (4.2-5.4)	5.5 (4.7-6.4)	$p = 0.002$
HF, median (IQR), n.u.	33.1 (26.0-38.1)	26.0 (22.3-39.9)	$p = 0.213$
LF/HF median (IQR)	1.9 (1.6-2.7)	2.8 (1.4-3.4)	$p = 0.187$
SD1, median (IQR), ms	21.2 (16.9-30.4)	27.5 (20.5-39.6)	$p = 0.010$
SD2, median (IQR), ms	27.2 (20.1-39.6)	42.1 (30.1-59.3)	$p < 0.001$
SD2/SD1, median (IQR)	1.2 (1.0-1.4)	1.4 (1.2-1.6)	$p = 0.049$
ApEn, median (IQR)	1.3 (1.2-1.4)	1.3 (1.1-1.4)	$p = 0.496$

ApEn = approximate entropy; HF = power in high-frequency range; LF = power in low-frequency range; NN50 = the number of pairs of successive NN (R-R) intervals that differ by more than 50 ms; pNN50 = the proportion of NN50 divided by the total number of NN (R-R) intervals; RMSSD = the square root of the mean squared differences of consecutive NN intervals; SDANN = the standard deviation of the average NN interval over short time divisions; SDNN = the standard deviation of all NN intervals; VLF = power in very-low-frequency range.
