# Supplementary material for: Heart Rate Variability in Acute Myocardial Infarction: Results of the HeaRt-V-AMI Single-Center Cohort Study
Source: J Cardiovasc Dev Dis. 2024 Aug 22;11(8):254. doi: 10.3390/jcdd11080254 (PMC11355001; doi:10.3390/jcdd11080254)
Supplement: Supplementary file 1 [file jcdd-11-00254-s001.zip › Table S3. Mortality correlation analysis.pdf]

Table S3. HRV parameters correlated significantly with in-hospital mortality.

HRV parameters	Spearman's rho	P-value
HRV during entire PCI duration		
LF, log	-0.194	p = 0.048
SD2/SD1	-0.262	p = 0.007
ApEn	-0.233	p = 0.017
HRV in the first 5 minutes of PCI		
SD2/SD1	-0.273	p = 0.005
HRV in the last 5 minutes of PCI		
SD2/SD1	-0.235	p = 0.016
ApEn = approximate entropy; HRV = heart rate variability; LF = power in low-frequency range; PCI = percutaneous coronary intervention		

HRV parameters	Deceased	Survivors	P-value
HRV during entire PCI duration			
SDNN, median (IQR), ms	23.9 (20.2-35.6)	29.2 (21.2-41.1)	p = 0.806
RMSSD, median (IQR), ms	38.9 (29.8-49.5)	32.3 (25.1-46.1)	p = 0.361
NN50, median (IQR), beats	102.0 (51.0-106.0)	86.5 (32.2-167.7)	p = 0.981
pNN50, median (IQR), %	14.1 (7.3-22.5)	7.2 (2.9-17.4)	p = 0.205
RR triangular index, median (IQR), ms	5.7 (4.9-6.3)	6.3 (5.3-8.8)	p = 0.447
VLF, median (IQR), ms^2	33.3 (29.2-76.2)	75.8 (37.7-142.6)	p = 0.155
VLF, median (IQR), log	3.5 (3.3-4.3)	4.3 (3.6-4.9)	p = 0.157
LF, median (IQR), ms^2	178.5 (113.5-194.5)	391.0 (178.2-681.7)	p = 0.064
LF, median (IQR), log	5.1 (4.7-5.2)	5.9 (5.1-6.5)	p = 0.052
LF, median (IQR), n.u.	63.8 (58.0-70.4)	66.9 (59.9-74.8)	p = 0.369
HF, median (IQR), ms^2	100.4 (81.6-106.4)	178.7 (96.5-323.5)	p = 0.108
HF, median (IQR), log	4.6 (4.4-4.6)	5.1 (4.5-5.7)	p = 0.106
HF, median (IQR), n.u.	35.9 (29.4-41.7)	32.7 (25.0-39.7)	p = 0.373
LF/HF median (IQR)	1.7 (1.3-2.3)	2.0 (1.5-2.9)	p = 0.352
SD1, median (IQR), ms	27.5 (21.1-35.1)	22.8 (17.7-32.6)	p = 0.349
SD2, median (IQR), ms	19.7 (19.2-36.3)	33.3 (22.7-45.2)	p = 0.240
SD2/SD1, median (IQR)	0.9 (0.8-1.0)	1.3 (1.0-1.6)	**p = 0.008**
ApEn, median (IQR)	1.2 (1.0-1.2)	1.3 (1.2-1.4)	**p = 0.019**
HRV in the first 5 minutes of PCI			
SDNN, median (IQR), ms	32.3 (20.5-44.8)	29.3 (21.0-43.5)	p = 0.841
RMSSD, median (IQR), ms	51.9 (32.0-69.0)	36.3 (24.3-51.5)	p = 0.240
NN50, median (IQR), beats	23.0 (17.0-33.0)	28.5 (11.0-50.7)	p = 0.847
pNN50, median (IQR), %	26.1 (7.4-26.6)	9.1 (2.8-20.5)	p = 0.174
RR triangular index, median (IQR), ms	5.1 (4.8-6.4)	6.2 (4.8-9.1)	p = 0.256
VLF, median (IQR), ms^2	24.0 (20.5-88.7)	66.0 (18.9-136.7)	p = 0.357
VLF, median (IQR), log	3.1 (3.0-4.4)	4.1 (2.9-4.8)	p = 0.365
LF, median (IQR), ms^2	139.7 (22.7-252.3)	387.5 (110.9-744.7)	p = 0.056
LF, median (IQR), log	4.9 (3.1-5.5)	5.8 (4.6-6.5)	p = 0.067
LF, median (IQR), n.u.	58.1 (53.1-59.8)	65.3 (55.0-73.8)	p = 0.252
HF, median (IQR), ms^2	93.3 (37.4-125.8)	194.7 (73.3-341.1)	p = 0.099
HF, median (IQR), log	4.5 (3.6-4.8)	5.2 (4.2-5.8)	p = 0.106
HF, median (IQR), n.u.	41.7 (39.9-46.6)	34.0 (25.8-44.3)	p = 0.216
LF/HF median (IQR)	1.3 (1.1-1.4)	1.9 (1.2-2.8)	p = 0.199
SD1, median (IQR), ms	36.7 (22.7-49.1)	25.7 (17.2-36.6)	p = 0.234
SD2, median (IQR), ms	27.2 (18.2-39.5)	32.7 (22.4-48.0)	p = 0.499
SD2/SD1, median (IQR)	0.8 (0.7-0.8)	1.2 (1.0-1.5)	**p = 0.006**
ApEn, median (IQR)	1.0 (0.6-1.0)	1.0 (0.9-1.1)	p = 0.188
HRV in the last 5 minutes of PCI			
SDNN, median (IQR), ms	24.3 (19.3-44.7)	28.4 (19.1-38.7)	p = 0.944
RMSSD, median (IQR), ms	34.2 (28.2-45.2)	29.8 (21.9-45.7)	p = 0.429
NN50, median (IQR), beats	39.0 (18.0-40.0)	18.0 (7.0-47.2)	p = 0.333
pNN50, median (IQR), %	10.2 (4.0-21.9)	4.5 (1.8-17.4)	p = 0.246
RR triangular index, median (IQR), ms	4.7 (4.3-7.1)	6.1 (4.5-8.0)	p = 0.580
VLF, median (IQR), ms^2	35.5 (18.7-77.9)	40.2 (23.2-155.0)	p = 0.489
VLF, median (IQR), log	3.5 (2.9-4.3)	3.69 (3.1-5.0)	p = 0.456
LF, median (IQR), ms^2	400.4 (81.8-424.4)	337.1 (106.5-794.2)	p = 0.377
LF, median (IQR), log	5.9 (4.4-6.0)	5.7 (4.6-6.6)	p = 0.352

LF, median (IQR), n.u.	72.9 (51.0-74.6)	65.1 (56.2-75.0)	p = 0.932
HF, median (IQR), ms^2	129.6 (78.3-143.6)	168.0 (59.3-328.7)	p = 0.279
HF, median (IQR), log	4.8 (4.3-4.9)	5.1 (4.0-5.6)	p = 0.262
HF, median (IQR), n.u.	26.8 (25.2-48.8)	34.4 (24.8-43.0)	p = 0.944
LF/HF median (IQR)	2.7 (1.0-2.9)	1.8 (1.3-2.9)	p = 0.938
SD1, median (IQR), ms	24.2 (19.9-32.0)	21.1 (15.5-32.4)	p = 0.429
SD2, median (IQR), ms	24.4 (18.8-33.6)	32.3 (21.4-46.4)	p = 0.329
SD2/SD1, median (IQR)	1.0 (0.9-1.0)	1.3 (1.0-1.6)	**p = 0.018**
ApEn, median (IQR)	1.0 (0.8-1.0)	1.0 (1.0-1.1)	p = 0.165
ApEn = approximate entropy; HF = power in high-frequency range; LF = power in low-frequency range; NN50 = the number of pairs of successive NN (R-R) intervals that differ by more than 50 ms; pNN50 = the proportion of NN50 divided by the total number of NN (R-R) intervals; RMSSD = the square root of the mean squared differences of consecutive NN intervals; SDANN = the standard deviation of the average NN interval over short time divisions; SDNN = the standard deviation of all NN intervals; VLF = power in very-low-frequency range.			
