# Supplementary material for: Heart Rate Variability in Acute Myocardial Infarction: Results of the HeaRt-V-AMI Single-Center Cohort Study
Source: J Cardiovasc Dev Dis. 2024 Aug 22;11(8):254. doi: 10.3390/jcdd11080254 (PMC11355001; doi:10.3390/jcdd11080254)
Supplement: Supplementary file 1 [file jcdd-11-00254-s001.zip › Table S4. HRV and MACE and VA.pdf]

Table S4. Significant differences in HRV parameters according to investigated outcomes.

HRV parameters	MACE	No MACE	P-value
HRV during entire PCI duration			
SD2/SD1, median (IQR)	0.95 (0.17)	1.3 (0.60)	p = 0.006
ApEn, median (IQR)	1.1 (0.20)	1.3 (0.20)	p = 0.005
HRV in the first 5 minutes of PCI			
SD2/SD1, median (IQR)	0.80 (0.15)	1.2 (0.50)	p = 0.003
HRV in the last 5 minutes of PCI			
SD2/SD1, median (IQR)	0.95 (0.17)	1.30 (0.50)	p = 0.006
**HRV parameters**	**VA**	**No VA**	**P-value**
HRV during entire PCI duration			
SD2/SD1, median (IQR)	1.0 (0.15)	1.3 (0.60)	p = 0.002
HRV in the first 5 minutes of PCI			
SD2/SD1, median (IQR)	0.85 (0.15)	1.30 (0.52)	p < 0.001
HRV in the last 5 minutes of PCI			
SD2/SD1, median (IQR)	1.0 (0.35)	1.40 (0.50)	p = 0.014
ApEn, median (IQR)	0.95 (0.22)	1.10 (0.10)	p = 0.015
ApEn = approximate entropy; HRV = heart rate variability; IQR = interquartile range; MACE = major adverse cardiovascular events; PCI = percutaneous coronary intervention; VA = ventricular arrhythmias			
