# Supplementary figures and images for: Heart Rate Variability in Acute Myocardial Infarction: Results of the HeaRt-V-AMI Single-Center Cohort Study
Source: J Cardiovasc Dev Dis. 2024 Aug 22;11(8):254. doi: 10.3390/jcdd11080254 (PMC11355001; doi:10.3390/jcdd11080254)

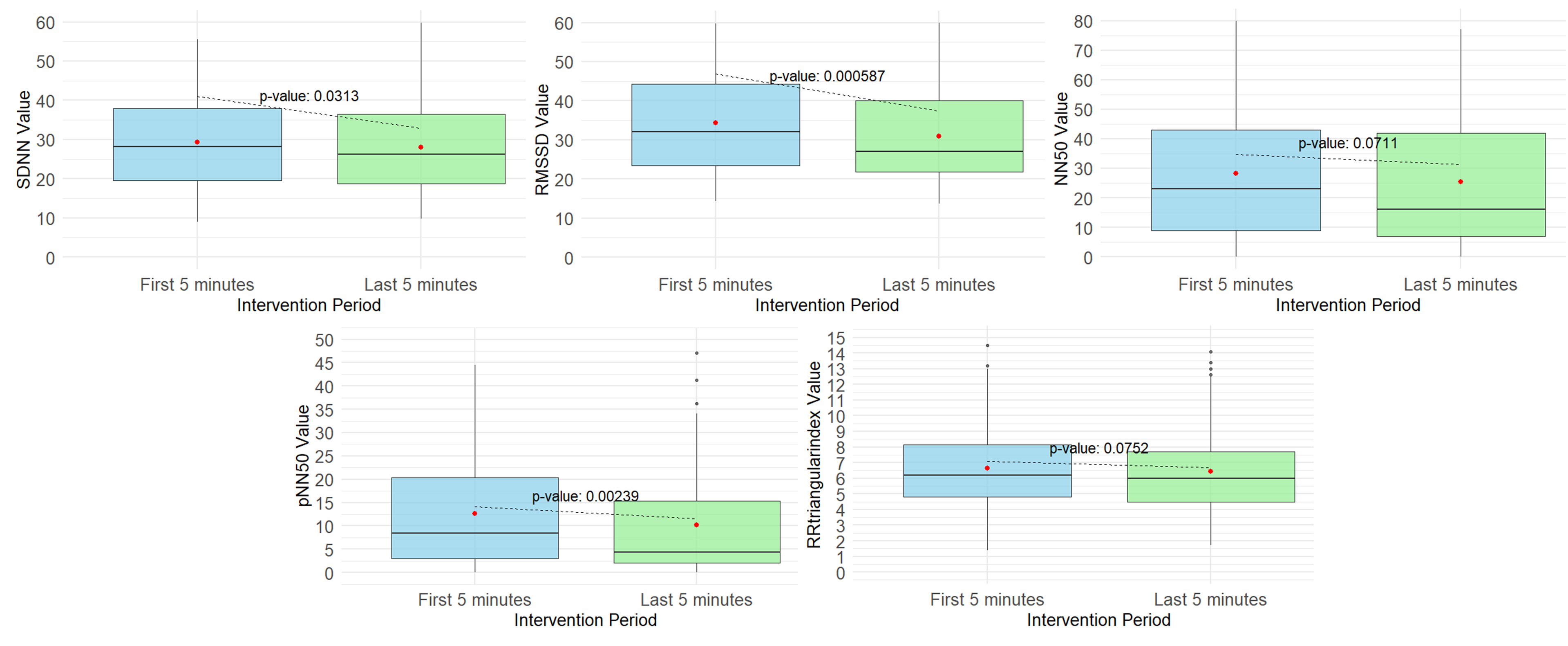

Supplement: Supplementary file 1 [file jcdd-11-00254-s001.zip › Figure S1. Time-domain parameters during PCI.tif]

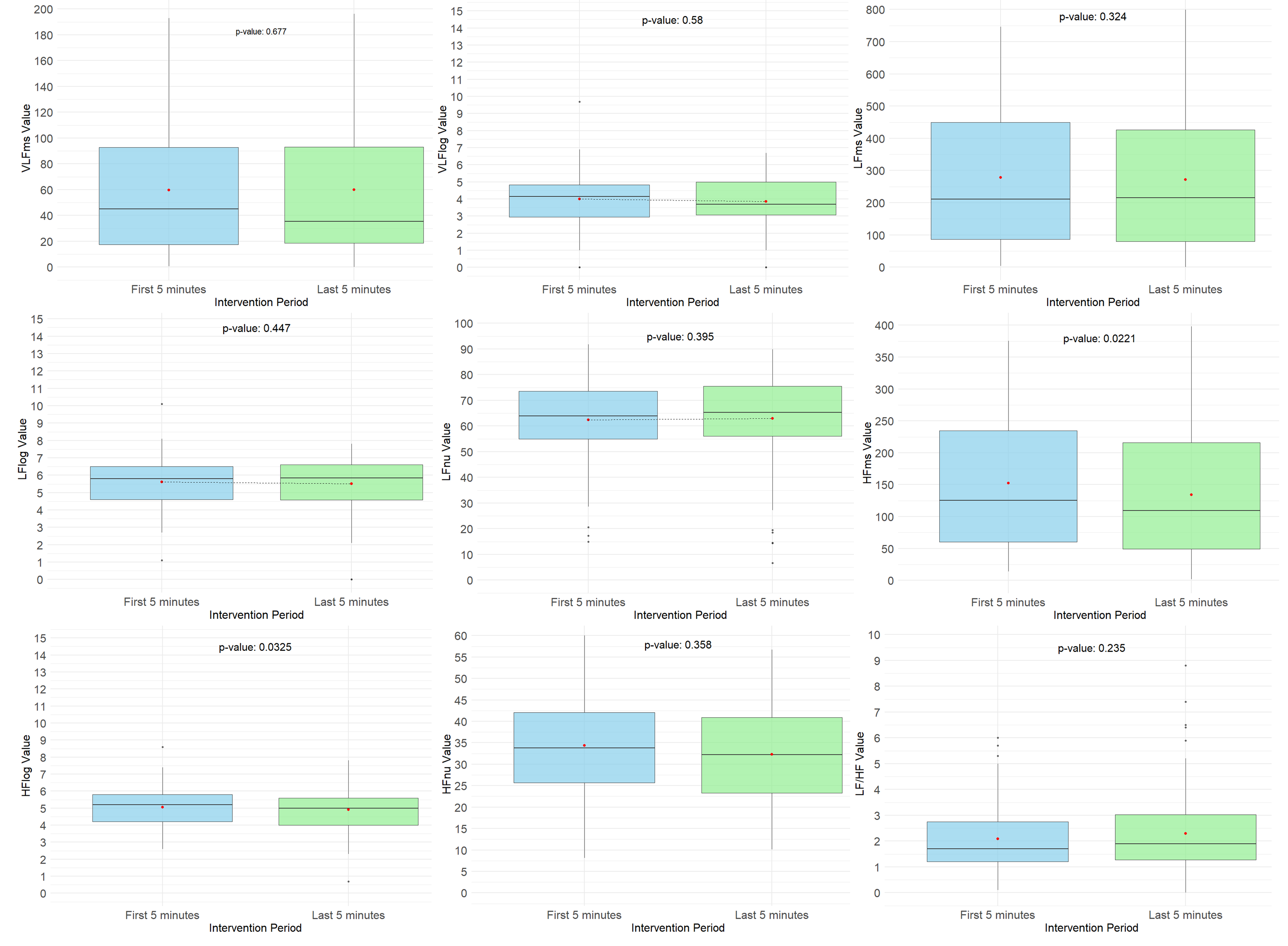

Supplement: Supplementary file 1 [file jcdd-11-00254-s001.zip › Figure S2. Frequency-domain parameters during PCI.tif]

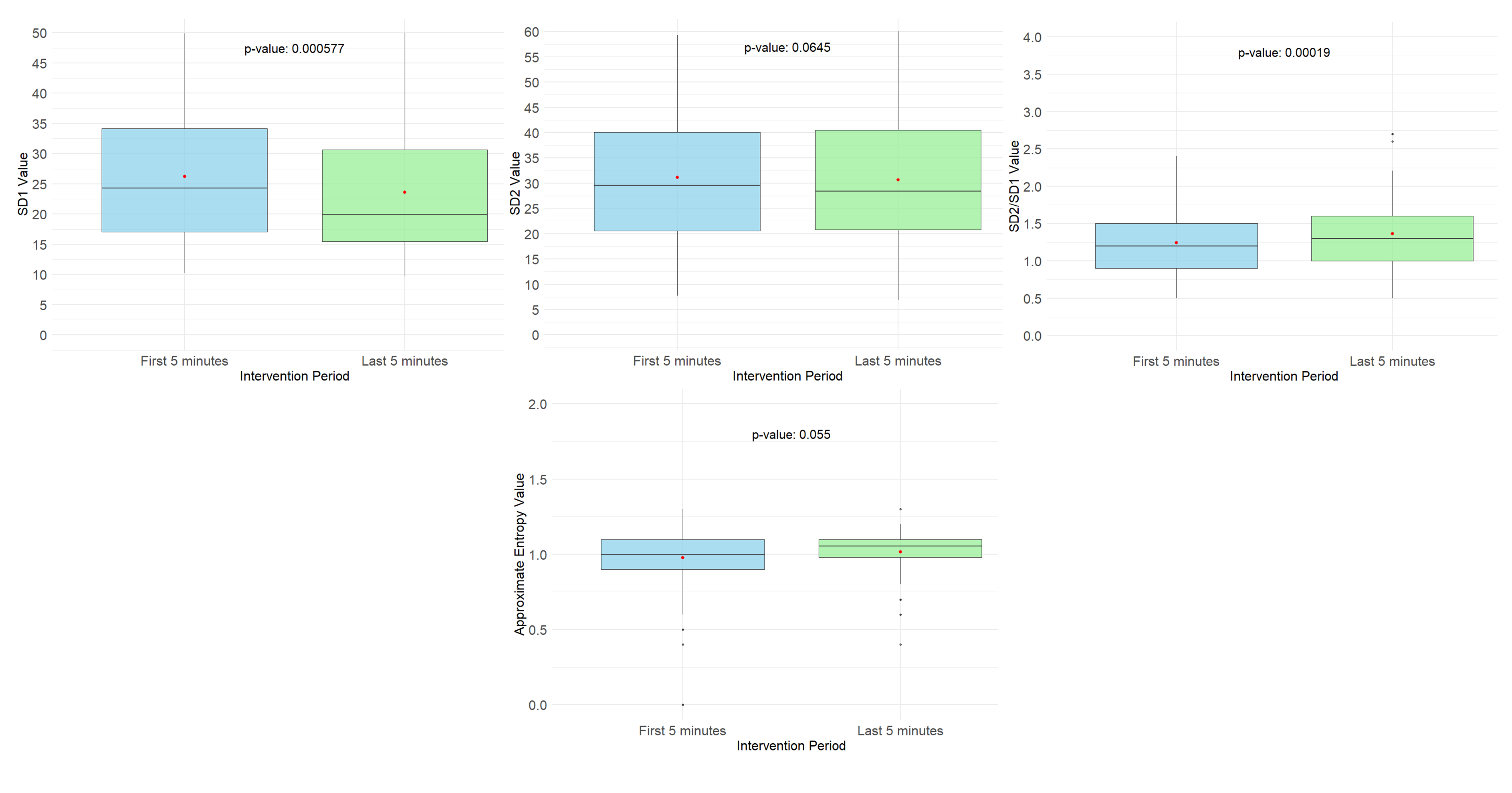

Supplement: Supplementary file 1 [file jcdd-11-00254-s001.zip › Figure S3. Non-linear parameters during PCI.tif]

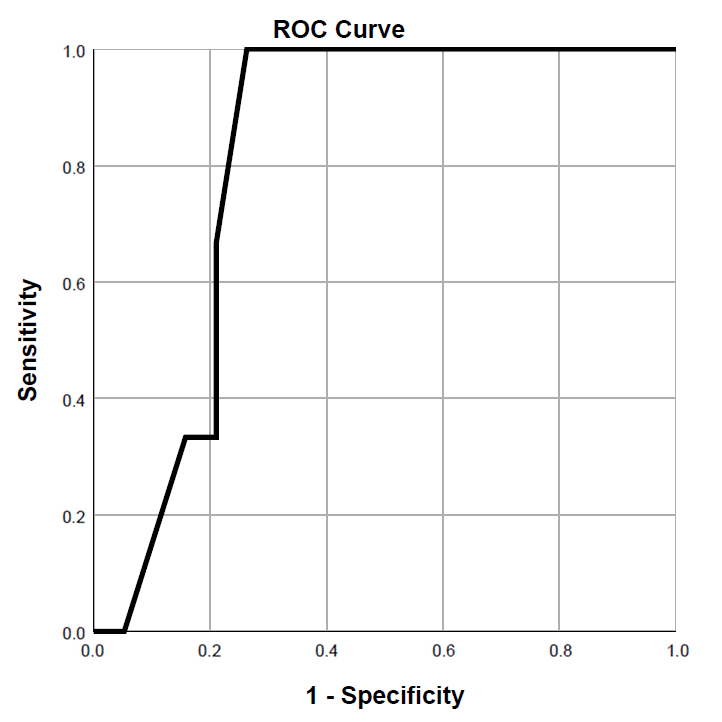

Supplement: Supplementary file 1 [file jcdd-11-00254-s001.zip › Figure S4. SD2SD1 and ApEn mortality low FE.png]
